# Supplementary material for: Job demands, job resources and postdoctoral job satisfaction: An empirical study based on the data from 2020 Nature global postdoctoral survey
Source: PLoS One. 2023 Nov 10;18(11):e0293653. doi: 10.1371/journal.pone.0293653 (PMC10637652; doi:10.1371/journal.pone.0293653)
Supplement: Attachment — Submitted filename: comments file.docx [file pone.0293653.s002.docx]

This paper examines the Job Demands,Job Resources, and Postdoctoral Job Satisfaction: An Empirical Study Based on the Data from Nature’s Global Postdoc Survey. The paper is in general not satisfactorily written and lacks novelty and justification for the need of study. I would like to make a few suggestions to improve the contributions of this research.

**Abstract**

The abstract should reflect the snapshot of the paper. It should be more persuasive. The Abstract lacks originality. Revisit the abstract specifically focusing on the following steps; (Brief glimpse into the topic, Purpose, Methodology, and Practical Implications).

**Introduction**

The Mechanism of introduction is not appropriate. It must include the territory of the study, niche, and detail about the current research, leading toward the need for this research. It lacks justification for; the need for the study, the selection of the variables, and the relevance of the population/context of the study. Furthermore, the introduction lacks to justify the contribution of this study to Academia and/or Industry, why is it worth studying? What`s new in it and why does it matter? In short, the entire introduction Part of the chapter needs serious attention.

**Literature Review**

- Under the heading "**1. Two-path hypothesis of the influence of job characteristics on post-doctoral job** **satisfaction”** It is mentioned that earlier, studies have been conducted on the same topic in the same context, then the question arises, “What the current study is going to add in it??
- A few references are NOT in APA style, as per the 7^th^ edition. See, For example, on page 10, the last line of the second paragraph.
- What do you mean by this term “In the university field? at page 10
- Hypothesis H4 is contradictory to Hypothesis H2. H2 states that job demands have a negative **indirect effect** on postdoctoral job satisfaction……. While H4 states that “Postdoctoral job satisfaction is highest with the combination of “**high demands**-high resources”. Means positively associated…?? Kindly elaborate on how??
- This section contains many grammatical mistakes and typo errors. Kindly proofread*.*
- The scholar has failed to develop a link between IVs and DV. A number of claims require referencing. Hence, the Literature requires significant rework.

**Research Methodology**

- Data collection methods, inclusion-exclusion criteria, Justification for sample size, etc. are not properly discussed.
- It is mentioned that “The right questions from the respective portions of the questionnaire were chosen to describe the variables being looked at". HOW??? Is any specific method applied?? Discuss its justification as well.
- No explanation of instrumentation is mentioned. E.g. job satisfaction questionnaire is adopted/adapted from?? Or was either developed??

**Statistical Methods and Results**

- Why SPSS is used?? And if SPSS, then why the quite older version is used since we have many revisions after SPSS 22.0? Instead, it is recommended to use SEM and SMART-PLS.
- Why two-factor ANOVA is used for mediation??? Can you provide any logical justification for it?
- “**Where the regression coefficients are explored in a two-path test with maximum likelihood estimation; and in the test of mediating and moderating effects using the self-help method (Bootstrap Method),** What is this? Where is the moderator in the entire study?? How mediation is checked through the self-help method? Etc.
- If SPSS, is used, then why not “Baron and Kenny” OR “Andrew Hayes approach” applied for mediation?
- **“The interaction hypothesis of job demands and job resources on postdoctoral job satisfaction”** What is the need for all this?? Elaborate on the justification of its relevance.
- **“Table 7 Self-help method test for the mediated role of the moderated”** What does this table indicate???
- What about the normality of data? Any test applied?? discuss
- The entire section does not have a single reference. The author is supposed to support its results from existing literature.
- In short, this section requires some serious attention.

**Discussion and Conclusion**

- Discussion should relate or contradict the study finding in light of existing research furthermore why your study found such results; there should be a discussion on results rather than just confirming the results.
- The conclusion of the study should be drafted in light of the objectives of the study. The author should highlight how each of the objectives identified in the research was attained.

**Final Evaluation Statement:**

The paper contains serious issues regarding, the novelty of the paper, justification of the study, methodology applied, and analysis section, besides, formatting issues like language, sequence, spelling, grammar, and coherence).
