# Supplementary material for: Job demands, job resources and postdoctoral job satisfaction: An empirical study based on the data from 2020 Nature global postdoctoral survey
Source: PLoS One. 2023 Nov 10;18(11):e0293653. doi: 10.1371/journal.pone.0293653 (PMC10637652; doi:10.1371/journal.pone.0293653)
Supplement: Attachment — Submitted filename: PLOS1 review report.docx [file pone.0293653.s004.docx]

I have completed the review of the article, and I can confirm that all the suggested changes have been diligently incorporated. However, there is one additional comment I would like to make here;

Please use the following article to justify the benchmark being met in confirmatory factor analysis for Cronbach's alpha, composite reliability, and AVE values:

Abboh, U. A., Majid, A. H., Fareed, M., & Abdussalaam, I. I. (2022). High-performance work practices lecturers’ performance connection: Does working condition matter? Management in Education, 0(0). <https://doi.org/10.1177/08920206211051468>
