# Supplementary material for: Job demands, job resources and postdoctoral job satisfaction: An empirical study based on the data from 2020 Nature global postdoctoral survey
Source: PLoS One. 2023 Nov 10;18(11):e0293653. doi: 10.1371/journal.pone.0293653 (PMC10637652; doi:10.1371/journal.pone.0293653)
Supplement: Attachment — Submitted filename: Response to Reviewers.docx [file pone.0293653.s005.docx]

**Revision Memo in response to Reviews of the Manuscript “Job Demands，Job Resources and Postdoctoral Job Satisfaction：An Empirical Study Based on the Data from 2020 Nature Global Postdoctoral Survey” PONE-D-23-15985**

We are very thankful to the editor for giving us chance to revise our paper. We also thank to all reviewers for their constructive feedback and comments to improve the quality of this paper. We have modified the paper according to the reviewers’ comments and provided line by line response to their comments and reference to changes in the revised paper. If the reviewer has new comments on the revised manuscript, we will revise it again.

**Replies to the comments of Reviewer # 1**

There is a minor issue in the interaction hypothesis development section. Like "H3b: Job resources can reduce the negative effect of job demands on postdoctoral burnout" is erroneously stated. It should be "Job resources can reduce the positive effect of job demands on postdoctoral burnout" as job demands are generally positively associated with the burnout outcomes.

Moreover, I would suggest to cite few lines from the discussion section of the paper referred below to further clarify the coping and gain spiral mechanism for your interaction hypothesis.

Mehboob, F., Othman, N., Fareed, M., & Raza, A. (2022). Change Appraisals and Job Crafting as Foundation to Inculcate Support for Change: A Dual Manifestation. Revista Brasileira de Gestão de Negócios, 24, 207-229.

**Reply:** We apologize for the mistake and and thank you for the correction. We have corrected H3b to "Job resources can reduce the positive effect of job demands on postdoctoral burnout". For details, please refer to L#34 of page 7.

Thank you for providing us with valuable references. We have supplemented the relevant content in the discussion section based on the references to clarify the interaction hypothesis. For details, please refer to L#21-24,33-37 of page 18.

**Replies to the comments of Reviewer # 2**

Please use the following article to justify the benchmark being met in confirmatory factor analysis for Cronbach's alpha, composite reliability, and AVE values:

Abboh, U. A., Majid, A. H., Fareed, M., & Abdussalaam, I. I. (2022). High-performance work practices lecturers’ performance connection: Does working condition matter? Management in Education, 0(0). https://doi.org/10.1177/08920206211051468

**Reply:** Thank you for providing us with valuable references. We have supplemented the relevant content in the result section based on the references to justify the benchmark being met . For details, please refer to L#10-11 of page 12, L#10-1 of page 13.
